# Mesenchymal Stromal Cells Suppress Hippocampal Neuron Autophagy Stress Induced by Hypoxic-Ischemic Brain Damage: The Possible Role of Endogenous IL-6 Secretion

**DOI:** 10.1155/2020/8822579

**Published:** 2020-08-28

**Authors:** Miao Yang, Wuqing Sun, Lu Xiao, Mulan He, Yan Gu, Ting Yang, Jie Chen, Xiaohua Liang

**Affiliations:** ^1^Children's Nutrition Research Center, Children's Hospital of Chongqing Medical University, Chongqing 400014, China; ^2^Chongqing Key Laboratory of Child Nutrition and Health, Chongqing 400014, China; ^3^Ministry of Education Key Laboratory of Child Development and Disorders, Chongqing 400014, China; ^4^China International Science and Technology Cooperation Base of Child Development and Critical Disorders, Chongqing 400014, China; ^5^Information Technological Service Center, Children's Hospital of Chongqing Medical University, Chongqing 400014, China

## Abstract

**Background:**

Increasing evidence has revealed that mesenchymal stromal cell (MSC) transplantation alleviates hypoxic-ischemic brain damage (HIBD) induced neurological impairments via immunomodulating astrocyte antiapoptosis effects. However, it remains unclear whether MSCs regulate neuron autophagy following HIBD.

**Results:**

In the present study, MSC transplantation effectively ameliorated learning-memory function and suppressed stress-induced hippocampal neuron autophagy in HIBD rats. Moreover, the suppressive effects of MSCs on autophagy were significantly weakened following endogenous IL-6 silencing in MSCs. Suppressing IL-6 expression also significantly increased p-AMPK protein expression and decreased p-mTOR protein expression in injured hippocampal neurons.

**Conclusion:**

Endogenous IL-6 in MSCs may reduce autophagy in hippocampal neurons partly through the AMPK/mTOR pathway.

## 1. Background

Hypoxic-ischemic brain damage (HIBD) in neonates may cause permanent brain damage, resulting in nervous system disability or even infantile mortality [1]. The hippocampus is easily damaged during the early stages of ischemia [2]. Necrosis, apoptosis, and autophagy are the main pathways of neuron death [3]. Recently, increased autophagy levels have been demonstrated following cerebral ischemia [4, 5]. It was reported that activation of autophagy after ischemia/reperfusion could be induced in neurons and astrocytes [6].

In the neuronal system, moderate autophagy is thought to be neuroprotective because it clears aggregated proteins associated with neurodegeneration, but both defective autophagy and excess autophagy may result in neuronal death [7–9]. More and more studies have demonstrated the involvement of autophagy in cerebral ischemic stroke; however, it remains unclear what effects transplanted mesenchymal stromal cells (MSCs) have on autophagy following ischemic cerebral injury.

Stem cells have potential biofunctions that induce tissue repair and regeneration. Numerous studies have demonstrated that MSC transplantation is neuroprotective in HIBD [10, 11]. MSCs could induce autolysosome formation and autophagy-dependent A*β* clearance in an Alzheimer's disease animal model to exert neuroprotective effects [12]. What is more, the results of our previous study indicated that IL-6 in the coculture medium was from MSCs, not injured neurons to play a neuroprotective role in HIBD rats [11].

Therefore, we speculated that the neuroprotective function of MSCs might partially regulate hippocampal autophagy in HIBD rats via IL-6 secretion. A series of experiments were designed to verify the above hypothesis. First, we evaluated the effects of MSCs on hippocampal neuron autophagy both *in vivo* and *in vitro*. Second, IL-6-silenced MSCs were used to verify the role of IL-6 in regulating autophagy. Finally, we tried to reveal the potential mechanisms of MSC-derived IL-6 to regulate hippocampal autophagy. This study may provide an experimental basis for the clinical application of MSCs.

## 2. Experimental Procedures

### 2.1. Animal Groups

Specific pathogen-free (SPF) grade Sprague Dawley (SD) rats (8 weeks old) were obtained from the Animal Center of Chongqing Medical University (Chongqing, China), and all animal experimental procedures followed the rules of the Animal Ethics Committee of Chongqing Medical University. The rats were fed in an SPF room at 25°C and 55-65% humidity with a 12 h light/dark cycle. At postnatal day 7, the pups were divided into a sham group (*n* = 24) and a HIBD group (*n* = 76) by the random number method. The HIBD group was subjected to HIBD injury as reported previously [13]. Briefly, the left carotid artery was ligated continuously, after two hours, the pups were then exposed to 8% oxygen at 37°C for 2.5 h. The pups in the sham group were subjected to only a cervical skin incision and subsequently sutured. The HIBD pups received intracerebroventricular transplants of 2 × 10^5^ MSCs, siIL-6 MSCs, or GFP MSCs in 5 *μ*l phosphate-buffered saline (PBS) (HyClone, USA) following HIBD for 30 min [14]. The intracerebroventricular injection was carried out 1.2 mm posterior to the bregma and 1.2 mm to the left of the lambdoid suture with a needle depth of 3.5 mm at a rate of 1 *μ*l/min for 5 min. The needle was kept in place for 2 min and then withdrawn slowly. The HIBD pups were injected with the same volume of PBS as the transplant negative control group. All of the operative SD rats were anesthetized at a dose of 40 mg/kg (intraperitoneal injection), and the concentration of pentobarbital sodium was 2%. To collect fresh hippocampal tissue, the rats were deeply anesthetized with 20% urethane at a dose of 1 g/kg (intraperitoneal injection), while the rats were performed to euthanasia by inhalation of CO_2_ at a 20% volume displacement per minute after the Morris water maze experiment.

### 2.2. Morris Water Maze

Four weeks after HIBD, the rats of the sham group (*n* = 10), the HIBD group (*n* = 10), and the HIBD+MSC group (*n* = 10) were evaluated for their spatial learning-memory functions with the Morris water maze task (MWM SLY-WMS 2.0, China) as previously described [15]. Briefly, the entire procedure was performed for six days. The first day, the rats' visual capabilities were assessed by visible platform tests, and from the 2^nd^ to 5^th^ days, the learning-memory function of the rats was trained with an invisible platform. On the 6^th^ day, a probe trial was performed without the platform, and the number of times that the rats crossed the former platform location in 60 s was recorded.

### 2.3. Preparation of MSCs and Treatment

Primary MSCs were isolated from rats and amplified with plastic adherence methods. The rat siIL-6-transduced recombinant lentivirus was constructed by NeuronBiotech Co., Ltd. The 4 different shRNA sequences were designed (GR425, GR426, GR427, and GR428) in a vector expressing green fluorescent protein (GFP), and the control sequence (TTCTCCGAACGTGTCACGT) served as a negative control (GFP MSCs) [16]. The siIL-6- and GFP-transduced recombinant lentiviruses were infected into the MSCs with virus titer of 3.47 × 10^8^ and IL-6 concentration was decreased by 70-80% [11].

### 2.4. Primary Hippocampal Neuron Injury

Primary cultures of hippocampal neurons were prepared from SD rats at embryonic days 17-18. The hippocampal neurons were cultured for 5 days in an incubator with 5% CO_2_ (Thermo, USA). For oxygen-glucose deprivation (OGD) injury, the hippocampal neurons were subjected to EBSS medium and exposed to 5% O_2_/5% CO_2_ for 1.5 h as described previously [17]; then, the EBSS was changed to standard neuronal culture medium. Cells cultured with standard neuronal culture medium in the presence of ambient (16%) O_2_/5% CO_2_ served as a control. The injured neurons were placed in Transwell inserts (Millipore, USA) for separate coculture with either (1) neural basal medium as a control or (2) MSCs subjected to the different treatments described above. After 12 h and 24 h, total protein was extracted using an extraction kit (BioTeke, China).

We found that the levels of Beclin 1 and LC3 II protein expressions were significantly increased in the rat hippocampus at 12-24 h following HIBD [18]. Meanwhile, OGD treatment upregulated autophagy-associated protein expression in primary neurons *in vitro* at 12 h.

### 2.5. Western Blotting

Total protein was extracted from the primary neurons and hippocampus for western blotting. The membranes were incubated in primary antibodies against Beclin 1 (1 : 1000, Abcam, USA), anti-LC3 II, anti-p62 (1 : 1000, Sigma, USA), anti-p-mTOR, anti-p-AMPK (1 : 1000, CST, USA), anti-IL-6 (1 : 500, R&D, China), and anti-*β*-actin (1 : 500, Santa Cruz, USA) at 4°C overnight. After incubation with HRP-conjugated secondary antibodies (Santa Cruz, USA) at room temperature for 1 h, the protein bands were developed using a chemiluminescent HRP substrate (Millipore, USA). Images were captured with a Syngene GBox Imaging System (Syngene, Europe Oxford, UK). The expression level of each protein was analyzed according to *β*-actin normalization.

### 2.6. Transmission Electron Microscopy

Primary neurons were digested with 0.1% trypsin and collected by centrifugation at 1200 rpm for 10 min. The neuron pellets were fixed in 4% glutaraldehyde and then postfixed in 1% osmium tetroxide. Following dehydration in a graded ethanol series, the samples were cut into ultrathin slices (40-60 nm thick), double stained with uranyl acetate and lead citrate, and observed by TEM (H-7500).

### 2.7. Statistical Analyses

Statistical analyses were performed using Statistical Product and Service Solutions 20 software. The values are presented as the mean ± standard error of the mean (SEM). Each experiment was repeated at least three times and analyzed by Student's *t*-test or one-way ANOVA with the least significant difference post hoc test. The escape latencies of the rats in the three groups were determined using ANOVA for repeated measurement. The least significant difference test was used to compare the mean of two or more groups. *P* < 0.05 was considered statistically significant.

## 3. Results

### 3.1. MSC Transplantation Downregulates Hippocampal Autophagy

**Figure 1:**
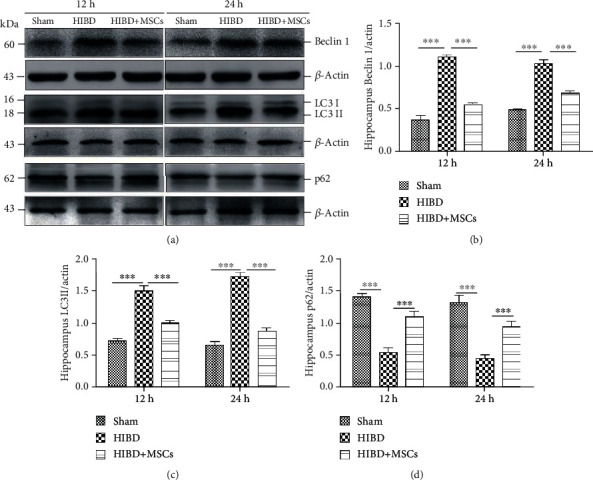
MSC transplantation reduced the expression levels of the autophagy-related proteins Beclin 1 and LC3 II and increased p62 expression levels in the hippocampus. (a) Representative western blots of Beclin 1, LC3 II, and p62 protein expressions in the hippocampus of the sham, HIBD, and HIBD+MSC groups following damage for 12 h and 24 h. (b–d) Quantification analysis of hippocampal protein expression levels normalized to *β*-actin. (*n* = 7, ^∗∗^*P* < 0.01, ^∗∗∗^*P* < 0.001).

Beclin 1 is a positive regulator of autophagy, LC3 II can reflect autophagical activity, and p62 is one of the selective substrates for autophagy. To evaluate the effect of MSCs on autophagy in the hippocampal neurons of neonatal rats with HIBD, we measured the autophagy-related proteins Beclin 1, LC3 II, and p62 in the hippocampus 12 h and 24 h after MSC transplantation. As shown in Figures 1(a) and 1(b), MSC transplantation significantly decreased Beclin 1 expression levels in the hippocampus of HIBD rats, whereas these levels were markedly increased in HIBD rats. The changes in LC3 II expression levels were highly consistent with those of Beclin 1 (Figures 1(a) and 1(c)). However, the levels of p62 protein expression were significantly increased in the HIBD hippocampus at 12 h and 24 h after MSC transplantation (Figures 1(a) and 1(d)). The above results demonstrated that MSC transplantation may regulate the level of hippocampal autophagy.

### 3.2. MSC Transplantation Alleviates Cognitive Impairment in HIBD Rats

**Figure 2:**
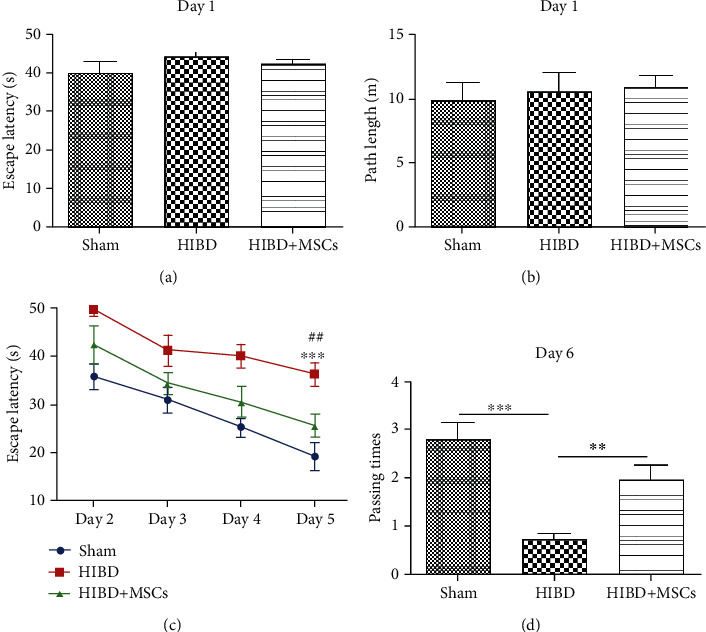
MSC transplantation improved the spatial learning-memory function of HIBD rats. (a, b) Escape latencies and path lengths to reach the visible platform for the sham, HIBD, and HIBD+MSC rats on the 1^st^ day of the MWM test. (c) Escape latencies of each group to locate the invisible platform from the 2^nd^ to the 5^th^ day in the MWM test (*n* = 10, ^∗∗∗^*P* < 0.001 vs. the sham group; ^##^*P* < 0.01 vs. the HIBD+MSC group). (d) Number of times passing through the former platform region for each group on the 6^th^ day of the MWM test (*n* = 10, ^∗∗∗^*P* < 0.001 vs. the sham group; ^∗∗^*P* < 0.01 vs. the HIBD+MSC group).

Impairment in learning-memory function is one of the major changes after HIBD. To confirm the effects of MSC transplantation on memory damage in HIBD rats, we conducted MWM tests. As shown in Figures 2(a) and 2(b), the escape latency and path length to locate the platform were not significantly different among the sham, HIBD, and HIBD+MSC groups on the first day, which indicates that neither HIBD nor MSC transplantation affected the motility or vision of the rats. During the training period with the hidden platform from the 2^nd^ to 5^th^ day, the escape latencies decreased progressively for all groups. However, the rats in the HIBD+MSC group spent less time locating the platform than those in the HIBD group but more time than those in the sham group (Figure 2(c)). Pairwise comparisons between different treatment groups showed statistically significant differences (^∗∗∗^*P* < 0.001 vs. the sham group; ^##^*P* < 0.01 vs. the HIBD+MSC group). The difference between days was significant (^∗∗∗^*P* < 0.001). There was no interaction between group and day (*P* > 0.05). In the spatial probe test on the 6^th^ day, the number of times passed through the original platform region was higher for HIBD+MSC rats than for HIBD rats, which passed through the original platform region the least number of times among the three groups (Figure 2(d)). These results suggest that MSC transplantation may regulate the level of hippocampal autophagy to alleviate memory impairment in HIBD rats.

### 3.3. MSC Coculture Reduces Autophagy and Decreases the Autophagosome Number in Primary Hippocampal Neurons following OGD

**Figure 3:**
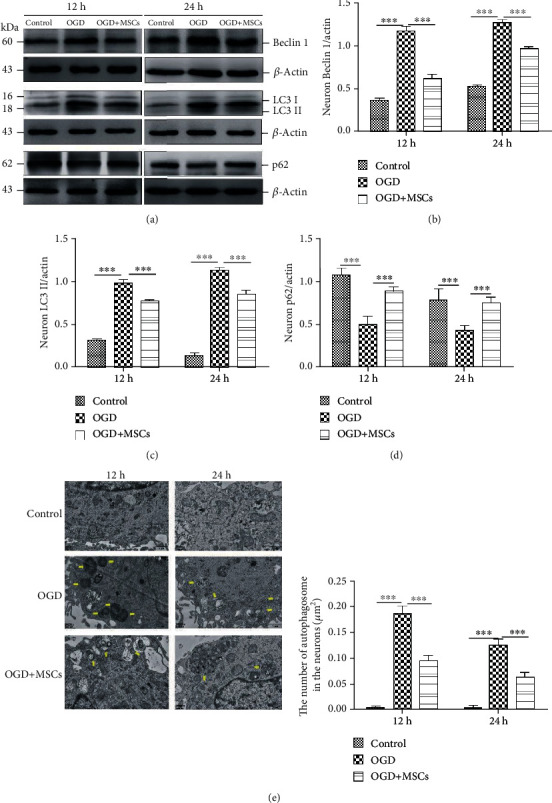
MSCs cocultured separately partly rescued the expression levels of the autophagy-associated proteins Beclin 1, LC3 II, and p62 and decreased the autophagosome numbers in primary hippocampal neurons with OGD injury. (a) Representative western blots of neuronal Beclin 1, LC3 II, and p62 protein expressions in the control, OGD, and OGD+MSC groups following injury for 12 h and 24 h. (b–d) Quantification analysis of the above protein expression levels normalized to *β*-actin in the primary neurons. (e) Number of autophagosomes in the neurons according to transmission electron microscopy (yellow arrows); quantification of the autophagosome numbers in the three groups. Scale bars = 2 *μ*m (*n* = 6, ^∗∗∗^*P* < 0.001).

To clarify the role of MSCs in regulating autophagy, we separately cocultured OGD-injured primary hippocampal neurons with MSCs. As shown in Figures 3(a)–3(d), OGD treatment obviously increased the Beclin 1 and LC3 II protein expression levels in primary neurons, while MSC-separated coculture significantly decreased Beclin 1 and LC3 II protein expression levels and induced p62 protein expression levels after OGD injury for 12 h and 24 h. These data were consistent with the changes *in vivo.* In addition, transmission electron microscopy was used to observe the numbers of autophagosomes in the OGD-damaged neurons following coculture with MSCs. The number of autophagosomes was significantly increased at 12 h and 24 h in the neurons with OGD injury. Interestingly, the increase in autophagosomes was decreased after separate MSC coculture (Figure 3(e)). The above results suggest that MSC-separated coculture could downregulate autophagy in neurons with OGD injury at the acute stage through paracrine secretion.

### 3.4. Silencing IL-6 Attenuated MSC Inhibition of Autophagy in OGD-Injured Neurons

**Figure 4:**
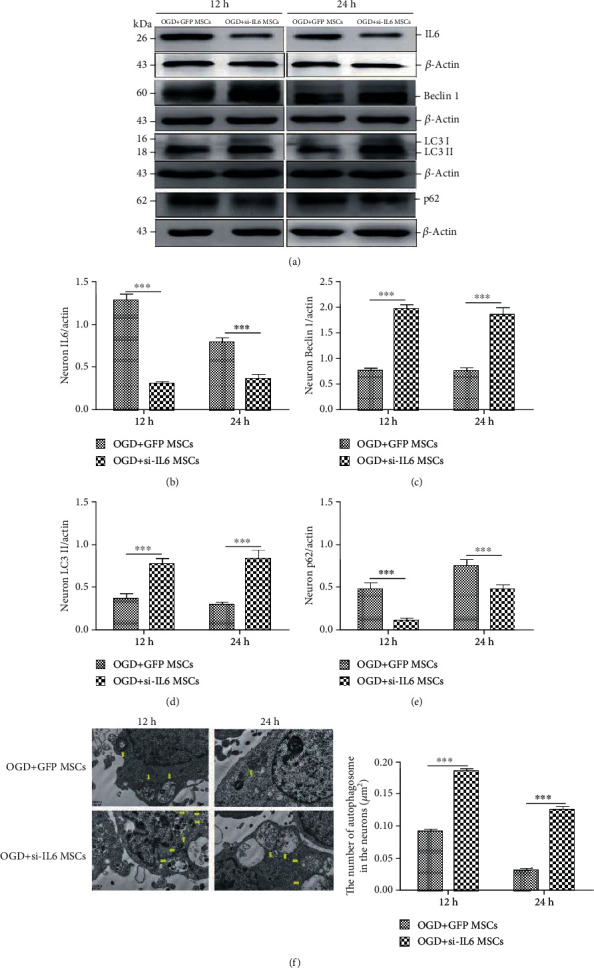
Separate siIL-6 MSC coculture increased Beclin 1 and LC3 II protein expression levels and reduced IL-6 and p62 protein expression levels, as well as increased the number of autophagosomes in hippocampal neurons injured by OGD, following damage for 12 h and 24 h. (a) Representative western blots of IL-6, Beclin 1, LC3 II, and p62 protein expression levels in the OGD-injured neurons cocultured with siIL-6 MSCs or GFP MSCs for 12 h and 24 h. (b–e) Quantification analysis of the neuron protein expression levels normalized to *β*-actin for the two groups. (f) Observation of autophagosomes in neurons from the two groups according to transmission electron microscopy (yellow arrows); quantification analysis of the autophagosome numbers in neurons from the two groups. Scale bars = 2 *μ*m, (*n* = 6, ^∗∗∗^*P* < 0.001).

Our previous study revealed that the neuroprotective function of MSCs was closely associated with IL-6 secretion and that siIL-6 lentivirus could effectively inhibit IL-6 release in MSCs [11]. To confirm the effect of endogenous IL-6 in MSCs on neuronal autophagy after injury, siIL-6 MSCs or GFP MSCs were cocultured with primary hippocampal neurons injured by OGD. As shown in Figures 4(a) and 4(b), the IL-6 levels at 12 h and 24 h after OGD were significantly lower in neurons with siIL-6 MSC-separated coculture than in those with GFP MSC coculture. Moreover, the expression levels of Beclin 1 and LC3 II were significantly higher in the OGD-damaged neurons following coculture with siIL-6 MSCs, and the p62 protein expression levels were significantly decreased (Figures 4(c)–4(e)). However, the number of autophagosomes at 12 h and 24 h was significantly increased in the siIL-6 MSC group compared with the GFP MSC group (Figure 4(f)). These results demonstrate that IL-6 secreted from MSCs could regulate autophagy in OGD-damaged neurons.

### 3.5. siIL-6 MSC Transplantation Weakened the Suppressive Effects of MSCs on Autophagy in the Hippocampus of HIBD Rats

**Figure 5:**
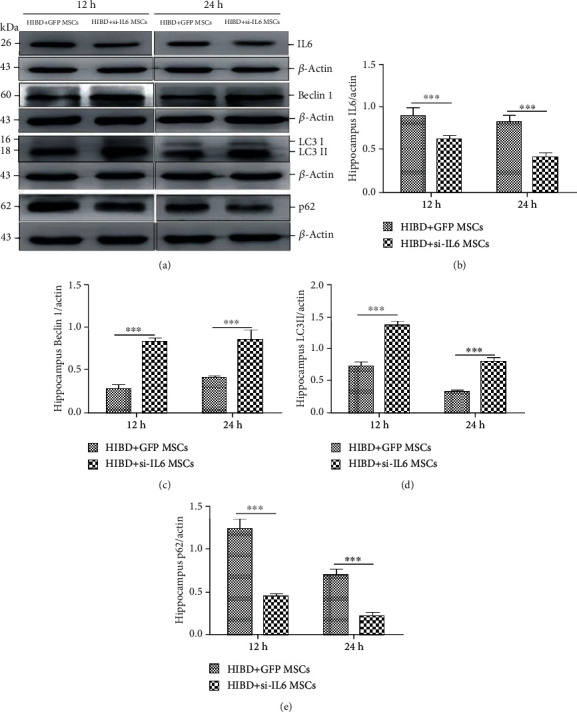
siIL-6 MSC transplantation impaired the effects of MSCs on Beclin 1, LC3 II, and p62 protein expression levels at 12 and 24 h after HIBD injury. (a) Representative western blots of hippocampal IL-6, Beclin 1, LC3 II, and p62 in HIBD rats following siIL-6 MSCs or GFP MSC transplantation for 12 h and 24 h. (b–e) Quantification analysis of hippocampal protein expression levels normalized to *β*-actin (*n* = 7, ^∗∗∗^*P* < 0.001).

When IL-6 expression levels were significantly decreased in the rat hippocampus following siIL-6 MSC transplantation (Figures 5(a) and 5(b)), the changes in Beclin 1, LC3 II, and p62 protein expressions completely mirrored the *in vitro* results (Figures 5(c)–5(e)). The results *in vivo* and *in vitro* indicate that endogenous IL-6 from MSCs can regulate autophagy in HIBD hippocampus neurons during the acute phase.

### 3.6. Effect of siIL-6 MSC Transplantation on AMPK/mTOR Signaling in the Rat Hippocampus after HIBD

**Figure 6:**
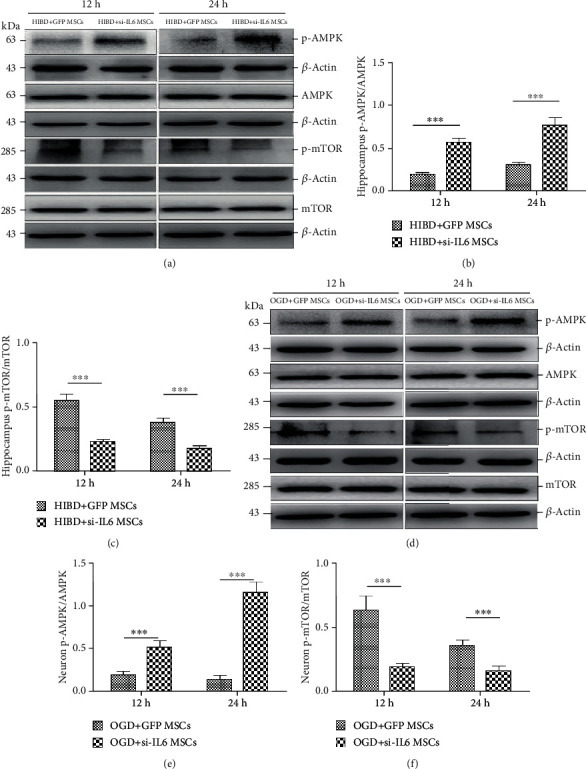
siIL-6 MSC transplantation increased hippocampal neuron p-AMPK protein expression levels and decreased p-mTOR levels following damage for 12 h and 24 h. (a) Representative western blots of hippocampal p-AMPK, AMPK, p-mTOR, and mTOR protein expression levels in the HIBD+siIL-6 MSC and HIBD+GFP MSC groups. (b, c) The ratio of p-AMPK/AMPK and p-mTOR/mTOR protein expression levels in the hippocampus (*n* = 7, ^∗∗∗^*P* < 0.001). (d) Representative western blots of the p-AMPK, AMPK, p-mTOR, and mTOR protein expression levels in the OGD+siIL-6 MSC and OGD+GFP MSCs group. (e, f) The ratio of p-AMPK/AMPK and p-mTOR/mTOR protein expression levels in the two groups (*n* = 6, ^∗∗∗^*P* < 0.001).

Furthermore, to explore the possible mechanism of IL-6-mediated autophagy, we investigated the AMPK/mTOR signaling pathway, which is downstream of IL-6 and involved in autophagy. Hippocampal p-AMPK protein expression levels were significantly increased, and p-mTOR protein expression levels were significantly reduced in the HIBD+siIL-6 MSC group compared with the HIBD+GFP MSC group (Figures 6(a)–6(c)). Similarly, p-AMPK expression levels were significantly higher, and p-mTOR levels were significantly lower in the OGD+siIL-6 MSC group than in the OGD+GFP MSC group (Figures 6(d)–6(f)). The above results suggest that IL-6 secretion from MSCs may inhibit the AMPK/mTOR signaling pathway to regulate autophagy in HIBD hippocampal neurons.

## 4. Discussion

The present study investigated whether MSCs regulate autophagy in hippocampal neurons to alleviate the learning-memory impairment of HIBD rats through endogenous IL-6 secretion. The current study demonstrated that IL-6 from MSCs reduced neuron autophagy by suppressing the AMPK/mTOR signaling pathway. This effect consequently improved the learning-memory function in HIBD rats.

Increasing evidence indicates that MSCs have potent therapeutic benefits for functional recovery after brain damage [11, 19], which improved overall neurobehavioral in both sensorimotor and cognitive testing [20]. In the present study, we also revealed that MSC transplantation ameliorated the learning-memory deficit in HIBD rats. The potential mechanisms of the MSC-induced neuroprotective effects following brain injury include cell replacement, trophic support from MSCs, immunomodulation, and endogenous brain restoration stimulation. An increasing number of studies have indicated the immunomodulatory function of MSCs in the damaged microenvironment after HIBD [19, 21, 22]. Some studies have shown that MSCs display neurorestorative effects through enhancing autolysosome formation to induce A*β* clearance in A*β*-treated AD models [12] or modulating a-synuclein expression in neurotoxin-treated Parkinson's disease (PD) models [23]. These findings suggest that MSCs play a protective role by regulating autophagy following injury, but autophagy regulation by MSCs in the hippocampal neurons of neonatal rats with HIBD has rarely been reported. According to our results, both MSC transplantation and separate coculture ameliorated the increased Beclin 1 and LC3 II expression levels and the decreased p62 protein levels induced by HIBD or OGD damage. Additionally, the number of autophagosomes in the neurons with OGD injury was reduced at the acute stage by MSCs coculture. Our results strongly indicate that MSCs suppress hippocampal neuron autophagy in HIBD rats, in contrast to the reports by Shin et al. and Park et al. [12, 23]. This difference is due to the different disease models, experimental animal ages, insult severity, ischemia stages, and autophagy degrees. Both PD and AD are chronic diseases of the nervous system, but the model used in our study is neonatal HIBD, which is an acute brain injury disease in the neonatal period.

In our previous study, we found that both transplanting MSCs and coculture *in vitro* could facilitate IL-6 release into the injured microenvironment. It was also shown that IL-6 in the damaged hippocampal microenvironment was primarily due to MSC transplantation, even though the IL-6 concentration was slightly increased after HIBD [11]. In the central nervous system (CNS), the IL-6 secretion level is low under normal conditions but is significantly induced under disease conditions [24]. However, the role of IL-6 in the damaged brain is controversial. IL-6 upregulation might increase harmful factors and mediate inflammatory cascades to effect the vascular endothelium and exacerbate cerebral ischemic damage [25]. However, IL-6 facilitates posttraumatic healing of the CNS via enhancing angiogenesis [26]. Both *in vivo* and *in vitro* results revealed that blocking IL-6 in MSCs significantly increases the levels of the autophagy-associated proteins Beclin 1 and LC3 II in both HIBD rat hippocampi and OGD-injured neurons; IL-6 silencing also reduced p62 protein expression levels. Moreover, the number of autophagosomes was significantly increased in the OGD-injured neurons following separate coculture with siIL-6 MSCs. These results are consistent with the findings of Chang et al. and Delk and Farach-Carson [27, 28], which demonstrate that endogenous IL-6 from MSCs can regulate hippocampal neuron autophagy following HIBD.

Mammalian AMPK has been confirmed to be a downstream target of IL-6. IL-6 can suppress mTOR, which is a pivotal factor in the autophagic signaling pathway, in an AMPK-dependent and STAT3-independent manner [29]. Under physiological and disease conditions, AMPK is activated by increased AMP and/or decreased ATP in the cytoplasm [30]. In this study, we found that IL-6 suppression significantly increased p-AMPK protein expression levels after siIL-6 MSC transplantation or coculture, suggesting that IL-6 in the damaged microenvironment can negatively regulate AMPK phosphorylation levels. Therefore, we speculated that IL-6 may inhibit the nonclassical AMPK pathway via the gp130-IL-6R receptor complex [27]. However, the details of this molecular mechanism require further study. mTOR is negatively regulated by AMPK signaling and plays multiple biological functions in the CNS, particularly in autophagy [31]. A decrease in the ATP concentration during ischemia activates AMPK, which subsequently suppresses mTOR activity to induce autophagy [32]. Our *in vivo* and *in vitro* observations indicate that phospho-mTOR protein expression levels were significantly decreased by siIL-6 MSCs. These results indicate that silencing IL-6 can suppress mTOR phosphorylation levels through p-AMPK activation. Active AMPK leads to the phosphorylation and activation of TSC1/2 and the inhibition of mTORC1 activity through Rheb [33]. The above findings demonstrate that MSCs suppress autophagy in hippocampal neurons to ameliorate the functional outcomes of HIBD, and this neuroprotective effect may partly involve the biofunction of endogenous IL-6 to reduce the AMPK/mTOR signaling pathway.

In the current study, we demonstrate the biological effect of endogenous IL-6 of mesenchymal stem cell on hippocampal autophagy after HIBD injury. Combined with our previous finding of IL-6 in MSCs facilitating antiapoptosis of injured astrocytes [11], we can conclude that MSC transplantation regulates injured microenvironment to ameliorate learning-memory dysfunction through both repressing neuron autophagy and enhancing antiapoptosis of astrocytes. Of course, our study had some limitations. The experimental data was mainly displayed in western blotting, no combined with the results of IHC or IF to confirm the regional change after injury with or without MSCs treatment, because the hypothesis of the present study was whether endogenous IL-6 of mesenchymal stem cell could suppress on hippocampal neuron autophagy after HIBD injury, rather than the localization of autophagy. Therefore, at first, we measured the levels of autophagy-associated protein expressions in the hippocampus of rats following HIBD with or without MSCs transplantation, as well as in the hippocampal neurons after OGD with or without MSCs coculture. Secondly, the autophagosomes were observed in the hippocampal primary neurons injured by OGD with or without MSCs or siIL-6-MSC coculture by transmission electron microscopy. In fact, autophagy occurs in all parts of the brain during the acute stage of HIBD injury, while our study only focused on hippocampal neuronal autophagy.

## 5. Conclusion

**Figure 7:**
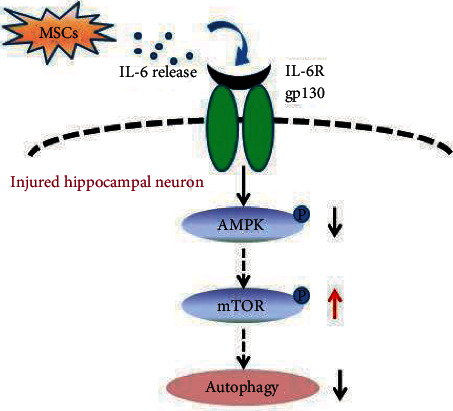
Summary diagram showing the role of IL-6 from MSCs in regulating hippocampal neuron autophagy in HIBD rats. In the current study, IL-6 from MSCs decreased p-AMPK protein expression levels to activate mTOR pathway phosphorylation, which in turn downregulated autophagy in the damaged hippocampal neurons.

This study revealed for the first time that endogenous IL-6 in MSCs may suppress autophagy in hippocampal neurons through inhibiting the AMPK/mTOR signaling pathway (Figure 7).

## Data Availability

The data used to support the findings of this study were supplied by Miao Yang and cannot be made freely available. Requests for access to these data should be made to [Miao Yang, 916844839@qq.com].

## Abbreviations

HIBD: Hypoxic-ischemic brain damage
OGD: Oxygen and glucose deprivation
MSCs: Mesenchymal stromal cells
IL-6: Interleukin-6
Beclin-1: Homologue of yeast Atg6
LC3: Microtubule-associated protein 1 light chain 3
P62: Sequestosome 1, SQSTM1
p-mTOR: Phospho-mammalian target of rapamycin
p-AMPK: Phospho-mammalian AMP-activated protein kinase
SD rats: Sprague Dawley rats
SPF: Specific pathogen-free
PBS: Phosphate-buffered saline
SEM: Standard error of the mean
ANOVA: One-way analysis of variance.
